# Racial and Ethnic Discrimination and Medical Students’ Identity Formation

**DOI:** 10.1001/jamanetworkopen.2024.39727

**Published:** 2024-10-16

**Authors:** Shruthi Venkataraman, Mytien Nguyen, Sarwat I. Chaudhry, Mayur M. Desai, Alexandra M. Hajduk, Hyacinth R. C. Mason, Alexis Webber, Dowin Boatright

**Affiliations:** 1 Department of Emergency Medicine, New York University Grossman School of Medicine, New York; 2 Department of Immunobiology, Yale School of Medicine, New Haven, Connecticut; 3 Section of General Internal Medicine, Department of Internal Medicine, Yale School of Medicine, New Haven, Connecticut; 4 Department of Chronic Disease Epidemiology, Yale School of Public Health, New Haven, Connecticut; 5 Section of Geriatrics, Department of Internal Medicine, Yale School of Medicine, New Haven, Connecticut; 6 Tufts University School of Medicine, Boston, Massachusetts; 7 Albany Medical Center, Albany, New York

## Abstract

**Importance:**

Investigating racial and ethnic discrimination in medical education is crucial for addressing disparities and fostering an inclusive environment.

**Objective:**

To assess how racial and ethnic discrimination in medical school is associated with personal and professional identity formation (PPIF) by race and ethnicity.

**Design, Setting, and Participants:**

This retrospective cross-sectional study used deidentified data on 37 610 medical students who matriculated in 2014 or 2015 and took the Association of American Medical Colleges Graduation Questionnaire (GQ) between 2016 and 2020. Statistical analysis was performed from September 1 to November 20, 2023.

**Exposures:**

Experiences of racial and ethnic discrimination were assessed through responses to 3 GQ questions about denial of opportunities, offensive remarks or names, and lower evaluations or grades due to race or ethnicity.

**Main Outcomes and Measures:**

Personal and professional development were measured as 2 separate outcomes using 2 GQ statements rated on a 5-point Likert scale (where 1 indicated strongly disagree and 5 indicated strongly agree): “My medical school has done a good job fostering and nurturing my development as a person” and “My medical school has done a good job fostering and nurturing my development as a physician.” Variables of personal and professional development were both dichotomized.

**Results:**

Of 37 610 medical students, 18 200 (48.4%) were female, and 19 410 (51.6%) were male; 2458 (6.5%) were African American or Black, 7801 (20.7%) were Asian, 2430 (6.5%) were Hispanic, 21 380 (56.9%) were White, 2404 (6.4%) were multiracial, and 1137 (3%) were other race or ethnicity. Most respondents attested that their medical school fostered their personal (27 272 [72.5%]) and professional (34 560 [91.9%]) development. African American or Black students reported the lowest rates of personal (1603 of 2458 [65.2%]) and professional (2182 of 2458 [88.8%]) development, and experienced lower likelihoods of personal (adjusted risk ratio [ARR], 0.89 [95% CI, 0.86-0.93]) and professional (ARR, 0.95 [95% CI, 0.94-0.97]) development than White students. Racial discrimination was inversely associated with development, with the highest PPIF rates among those never experiencing discrimination (personal, 25 089 of 33 508 [74.9%]; and professional, 31 257 of 33 508 [93.3%]). Those experiencing isolated discrimination (personal: ARR, 0.83 [95% CI, 0.80-0.87]; professional: ARR, 0.92 [95% CI, 0.91-0.95]) and recurrent discrimination (personal: ARR, 0.63 [95% CI, 0.60-0.66]; professional: ARR, 0.82 [95% CI, 0.80-0.84]) had relatively lower likelihoods of PPIF. African American or Black students experienced the highest rate of recurrent discrimination (543 of 2458 [22.1%]). No significant PPIF risk differences were found for other racial and ethnic groups underrepresented in medicine without discrimination compared with White students without discrimination, but all groups with recurrent discrimination had relatively lower PPIF risk.

**Conclusions and Relevance:**

In this cross-sectional study of US medical students, racial and ethnic discrimination was associated with lower PPIF across all racial and ethnic groups compared with White students without such experiences. African American or Black students disproportionately faced this discrimination. Systemic changes in medical education are needed to combat discrimination and ensure equity in holistic student development.

## Key Points

**Question:**

Is racial and ethnic discrimination associated with personal and professional development in medical school?

**Findings:**

In a cross-sectional study of 37 610 medical students, a higher frequency of racial and ethnic discrimination was significantly associated with diminished personal and professional development fostered by medical schools among all students. African American or Black students experienced racial and ethnic discrimination significantly more frequently than their peers from other racial and ethnic backgrounds.

**Meaning:**

This study suggests that racial and ethnic discrimination is associated with personal and professional identity formation in medical school, underscoring the need for targeted interventions to address discrimination in medical education, particularly to support the holistic development of African American or Black learners.

## Teaser

This cross-sectional study assesses how racial and ethnic discrimination in medical school is associated with personal and professional development of medical students.

## Introduction

The Association of American Medical Colleges (AAMC) underscores personal and professional development, or the acquisition of qualities to sustain lifelong growth as a person and physician, as foundational competencies to be instilled by medical training.^1^ The learning environment in medical school contributes to shaping professional identity formation and is influenced by interactions with peers, patients, and supervisors.^2,3^

The medical school learning environment, however, is not immune to the broader societal issues of racial and ethnic discrimination, particularly affecting students from ethnoracial backgrounds that are underrepresented in medicine.^4,5^ Experiences of racial and ethnic discrimination influence wellness and success in medical school and are associated with depression,^6^ burnout,^7^ and increased attrition rates.^8^ Emerging evidence suggests that subtle acts of racial and ethnic bias in the clinical learning environment can hinder professional identity formation among medical students from racial and ethnic minority groups.^9^ These experiences are alienating,^9^ leading to feelings of discomfort and invisibility, and require constant vigilance,^10^ potentially contributing to a deleterious learning climate.

Despite the existing research, no studies to date have broadly explored how the experience of racial and ethnic discrimination may be associated with personal and professional identity formation (PPIF) among medical students from diverse racial and ethnic backgrounds. This study addresses this knowledge gap by using a national cohort of allopathic US medical students to examine the association of racial and ethnic discrimination with PPIF.

## Methods

### Data and Participants

Using deidentified student-level data from the AAMC data warehouse and the American Medical College Application Service, we conducted a retrospective cross-sectional study of all allopathic doctor of medicine matriculants from the 2014-2015 and 2015-2016 academic years who graduated between 2016 and 2020 and took the Graduation Questionnaire (GQ). Written informed consent was obtained by the AAMC for students who responded to the GQ. Deidentified data were then provided to the study team by the AAMC. This study was deemed exempt by the Yale School of Medicine institutional review board because the data were deidentified. Our study followed the Strengthening the Reporting of Observational Studies in Epidemiology (STROBE) reporting guideline for cross-sectional studies.

### Student Sociodemographic Characteristics

Race and ethnicity data were provided by the AAMC for US citizens and permanent residents. Students self-reported their race and ethnicity as corresponding to the following groups: Hispanic, Latino, or of Spanish origin; American Indian or Alaska Native; African American or Black; Asian; Hawaiian Native or Other Pacific Islander; White; other; and unknown. Students who reported their race and ethnicity as corresponding to 2 or more of these groups were categorized as multiracial. For all analyses, participants who self-identified as American Indian or Alaska Native, Hawaiian Native or Other Pacific Islander, or other were grouped together as “other,” given the low sample size in the American Indian or Alaska Native and Hawaiian Native or Other Pacific Islander groups.

Experience of racial and ethnic discrimination was assessed via the following 3 questions in the GQ: (1) “During medical school, how frequently have you been denied opportunities for training or rewards based on race or ethnicity?” (2) “During medical school, how frequently have you been subjected to racially or ethnically offensive remarks/names?” (3) “During medical school, how frequently have you received lower evaluations or grades solely because of race or ethnicity rather than performance?” To each question, participants could respond “never,” “once,” “occasionally,” or “frequently.” We recategorized the variable such that participants who responded “once” to only 1 of the questions were labeled as having isolated experiences of racial and ethnic discrimination and those who responded either “occasionally,” “frequently,” or “once” to more than 1 question were labeled as having recurrent experiences of racial and ethnic discrimination.

### Outcomes

Personal and professional identity formation was assessed in 2 domains: personal and professional development. Personal development was assessed via the GQ item “My medical school has done a good job of fostering and nurturing my development as a person,” and professional development was assessed via the GQ item “My medical school has done a good job of fostering and nurturing my development as a physician.” Answers were gauged via a 5-point Likert scale where 1 indicated strongly disagree and 5 indicated strongly agree. We dichotomized both PPIF variables such that only participants who expressed strong agreement (ie, “agree” or “strongly agree”) were considered as reporting that their medical school nurtured their identity formation.

### Statistical Analysis

Statistical analysis was performed from September 1 to November 20, 2023. All data analyses were conducted using Stata, version 16.1 (StataCorp LLC). Missing data were present across several key variables, with the extent of missingness varying. Specifically, data for 2429 students (6.5%) were missing for race and ethnicity, 7925 (21.1%) for experience of racial and ethnic discrimination, 7750 (20.6%) for personal development, and 7459 (19.8%) for professional development. A total of 27 449 participants answered the GQ questions relevant to this study. Missing data were imputed using a fully conditional specification method to handle arbitrary missing patterns across all categorical data. The imputation model included all sociodemographic variables and PPIF outcome variables. Twenty imputed datasets were created using the mi impute chained command in Stata. Summarization across sets was done using mi estimate.

To investigate the independent associations of race and ethnicity, and experience of racial and ethnic discrimination, with PPIF, we used generalized linear models with a Poisson distribution and robust SEs^11,12,13^ wherein race and ethnicity or experience of racial and ethnic discrimination were independent variables and PPIF was the outcome variable. To assess whether experience of racial and ethnic discrimination mediates the association of race and ethnicity with PPIF, we stratified race and ethnicity based on the level of racial and ethnic discrimination experience and conducted generalized linear models with a Poisson distribution and robust SEs. All generalized linear models were adjusted for sex, to account for sex-related differences in PPIF (eTable 2 in Supplement 1). Sensitivity analyses showed consistent findings between complete-case analysis and multiple imputation, and imputation resulted in a better-fitting model, so only the latter results are reported. Two-sided *P* < .05 indicated statistical significance.

## Results

The final sample included 37 610 students. Female students constituted 48.4% of the sample (n = 18 200) and male students 51.6% (n = 19 410) (Table). The sample included 2458 African American or Black students (6.5%), 7801 Asian students (20.7%), 2430 Hispanic students (6.5%), 21 380 White students (56.9%), 2404 multiracial students (6.4%), and 1137 students of other race or ethnicity (3.0%). Most students reported that their medical school fostered their personal development (72.5% [n = 27 272]) and professional development (91.9% [n = 34 560]). Although 89.1% (n = 33 508) of participants had never experienced racial and ethnic discrimination, 4.3% (n = 1634) had isolated incidents and 6.6% (n = 2468) experienced recurrent incidents.

**Table.** Participant Demographic Characteristics

Characteristic^a^	Dataset participants, No. (%)	
	Original (N = 37 610)	Imputed (n = 37 610)
Sex		
Female	18 200 (48.4)	18 200 (48.4)
Male	19 410 (51.6)	19 410 (51.6)
Race and ethnicity		
African American or Black	2290 (6.1)	2458 (6.5)
Asian	7299 (19.4)	7801 (20.7)
Hispanic, Latino, or of Spanish origin	2275 (6.0)	2430 (6.5)
White	20 003 (53.2)	21 380 (56.9)
Multiracial	2253 (6.0)	2404 (6.4)
Other^a^	1061 (2.8)	1137 (3.0)
Missing	2429 (6.5)	NA
Experience of racial and ethnic discrimination		
Never	26 438 (70.3)	33 508 (89.1)
Isolated	1311 (3.5)	1634 (4.3)
Recurrent	1936 (5.1)	2468 (6.6)
Missing	7925 (21.1)	NA
Personal development		
Yes	21 644 (57.5)	27 272 (72.5)
No	8216 (21.9)	10 338 (27.5)
Missing	7750 (20.6)	NA
Professional development		
Yes	27 705 (73.7)	34 560 (91.9)
No	2446 (6.5)	3050 (8.1)
Missing	7459 (19.8)	NA

Abbreviation: NA, not applicable.

^a^ Participants who self-identified as American Indian or Alaska Native, Hawaiian Native or Other Pacific Islander, or other were grouped together as “other,” given the low sample size in the American Indian or Alaska Native and Hawaiian Native or Other Pacific Islander groups.

### Association of Race and Ethnicity With PPIF in Medical Education

**Figure 1.**
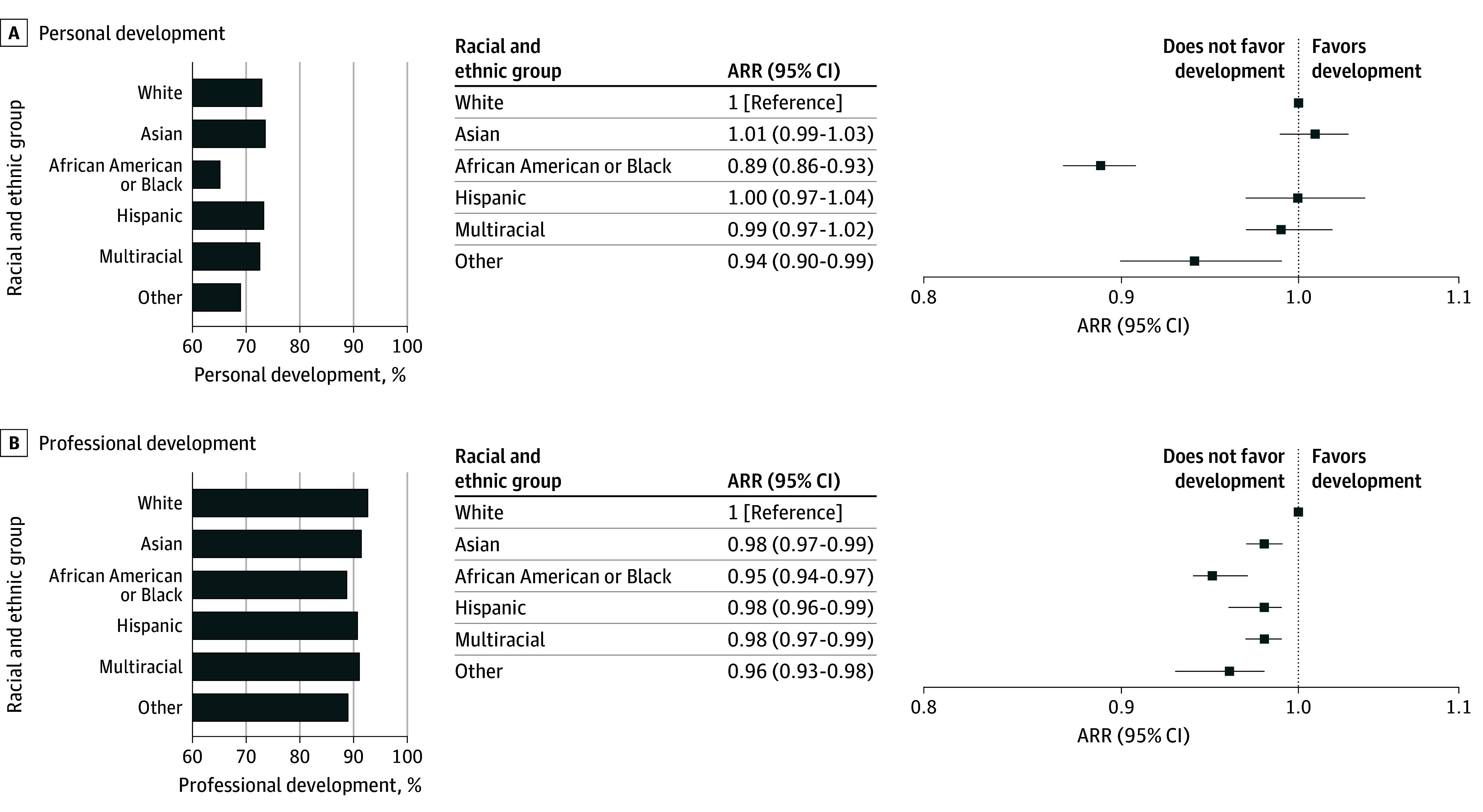
Personal and Professional Development Among Medical School Students by Race and Ethnicity The bar graphs show the proportion of students in each racial and ethnic group reporting that their medical school fostered their personal (A) and professional (B) development. The associated forest plots illustrate sex-adjusted risk ratios (ARRs) for personal and professional development, comparing all racial and ethnic groups with the White reference group.

African American or Black students reported the lowest level of agreement (65.2% [1603 of 2458]) that their medical school fostered and nurtured their personal development, followed by multiracial students (72.6% [1745 of 2404]), White students (73% [15 617 of 21 380]), Hispanic students (73.3% [1782 of 2430]), and Asian students (73.6% [5740 of 7801]) (χ^2^_5_ = 80.9; *P* < .001; Figure 1). Compared with White students, African American or Black students were less likely to report their medical school fostering their personal development (sex-adjusted risk ratio [ARR], 0.89 [95% CI, 0.86-0.93]). No significant differences were found in this outcome between White students and their Asian (ARR, 1.01 [95% CI, 0.99-1.03]), Hispanic (ARR, 1.00 [95% CI, 0.97-1.04]), or multiracial (ARR, 0.99 [95% CI, 0.97-1.02]) counterparts.

African American or Black students also reported the lowest level of agreement (88.8% [2182 of 2458]) that their medical school fostered and nurtured their professional development, followed by Hispanic students (90.8% [2206 of 2430]), multiracial students (91.2% [2193 of 2404]), Asian students (91.5% [7136 of 7801]), and White students (92.7% [19 829 of 21 380]) (χ^2^_5_ = 72.2; *P* < .001; Figure 1). Similar trends were observed for professional development as for personal development, with African American or Black students (ARR, 0.95 [95% CI, 0.94-0.97]) less likely to report that their medical school fostered their professional development relative to White students.

### Association of Racial and Ethnic Discrimination Experience With PPIF in Medical Education

**Figure 2.**
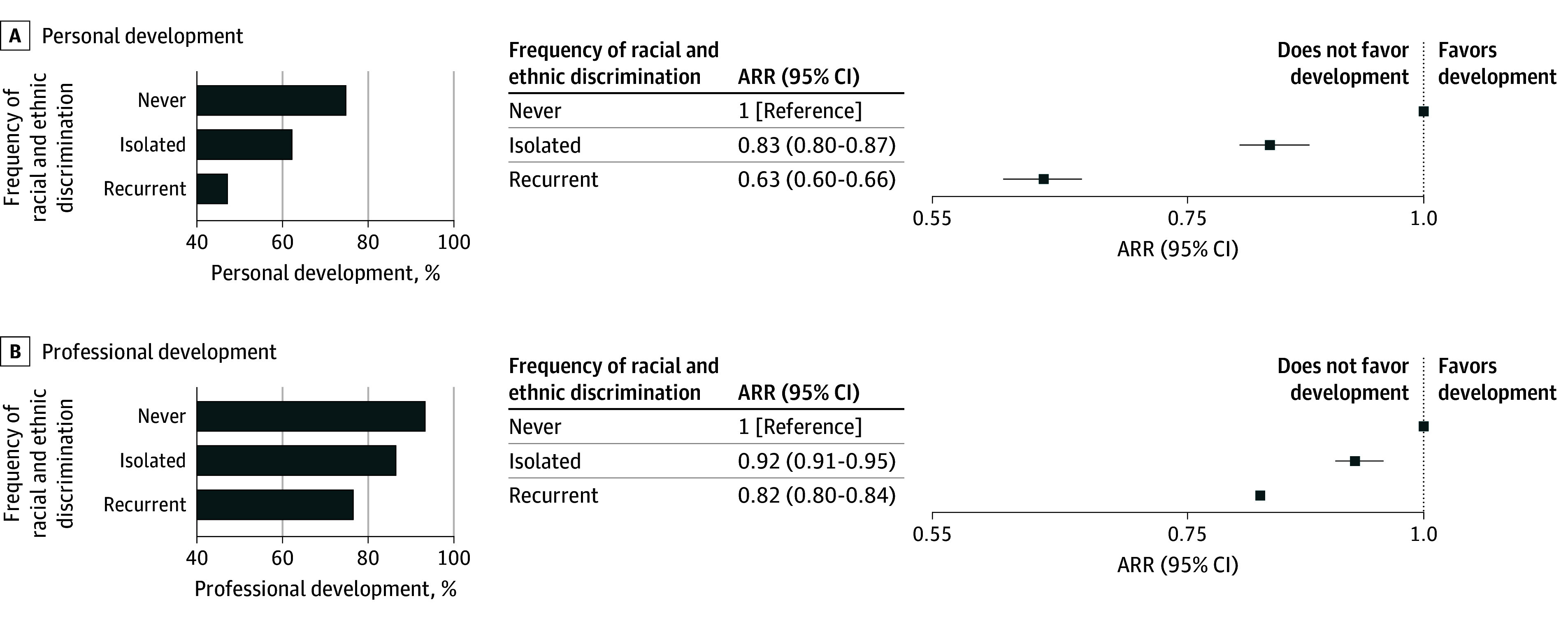
Association of Racial and Ethnic Discrimination With Personal and Professional Development The bar graphs show the proportion of students with no, isolated, and recurrent experiences of racial and ethnic discrimination reporting that their medical school fostered their personal (A) and professional (B) development. The associated forest plots display the sex-adjusted risk ratios (ARRs) for personal and professional development, indicating the worsening negative association of racial discrimination experience with medical schools’ ability to foster personal and professional development.

Participants who had never experienced racial and ethnic discrimination reported the highest level of agreement that their medical school fostered their personal development (74.9% [25 089 of 33 508]), followed by those who experienced isolated incidences of racial and ethnic discrimination (62.3% [1018 of 1634]) and those who experienced recurrent incidences of racial and ethnic discrimination (47.2% [1166 of 2468]) (χ^2^_2_ = 969.9; *P* < .001; Figure 2). After adjustment for sex, individuals with isolated experiences of racial and ethnic discrimination were less likely to report that their medical school fostered their personal development (ARR, 0.83 [95% CI, 0.80-0.87]), and those with recurrent experiences were considerably less likely (ARR, 0.63 [95% CI, 0.60-0.66]), compared with those who had never experienced racial and ethnic discrimination.

Similar trends were observed for professional development. Participants who had never experienced racial and ethnic discrimination reported the highest level of agreement that their medical school fostered and nurtured their professional development (93.3% [31 257 of 33 508]), followed by those who experienced isolated incidences of racial and ethnic discrimination (86.5% [1413 of 1634]) and those who experienced recurrent incidences of racial and ethnic discrimination (76.6% [1890 of 2468]) (χ^2^_2_ = 927.7; *P* < .001; Figure 2). After adjusting for sex, we found that individuals with isolated experiences of racial and ethnic discrimination were less likely to report that their medical school fostered their professional development (ARR, 0.92 [95% CI, 0.91-0.95]), and those with recurrent experiences were even more unlikely to do so (ARR, 0.82 [95% CI, 0.80-0.84]) compared with those who had never experienced racial and ethnic discrimination.

### Racial and Ethnic Discrimination and the Association of Race and Ethnicity With PPIF

**Figure 3.**
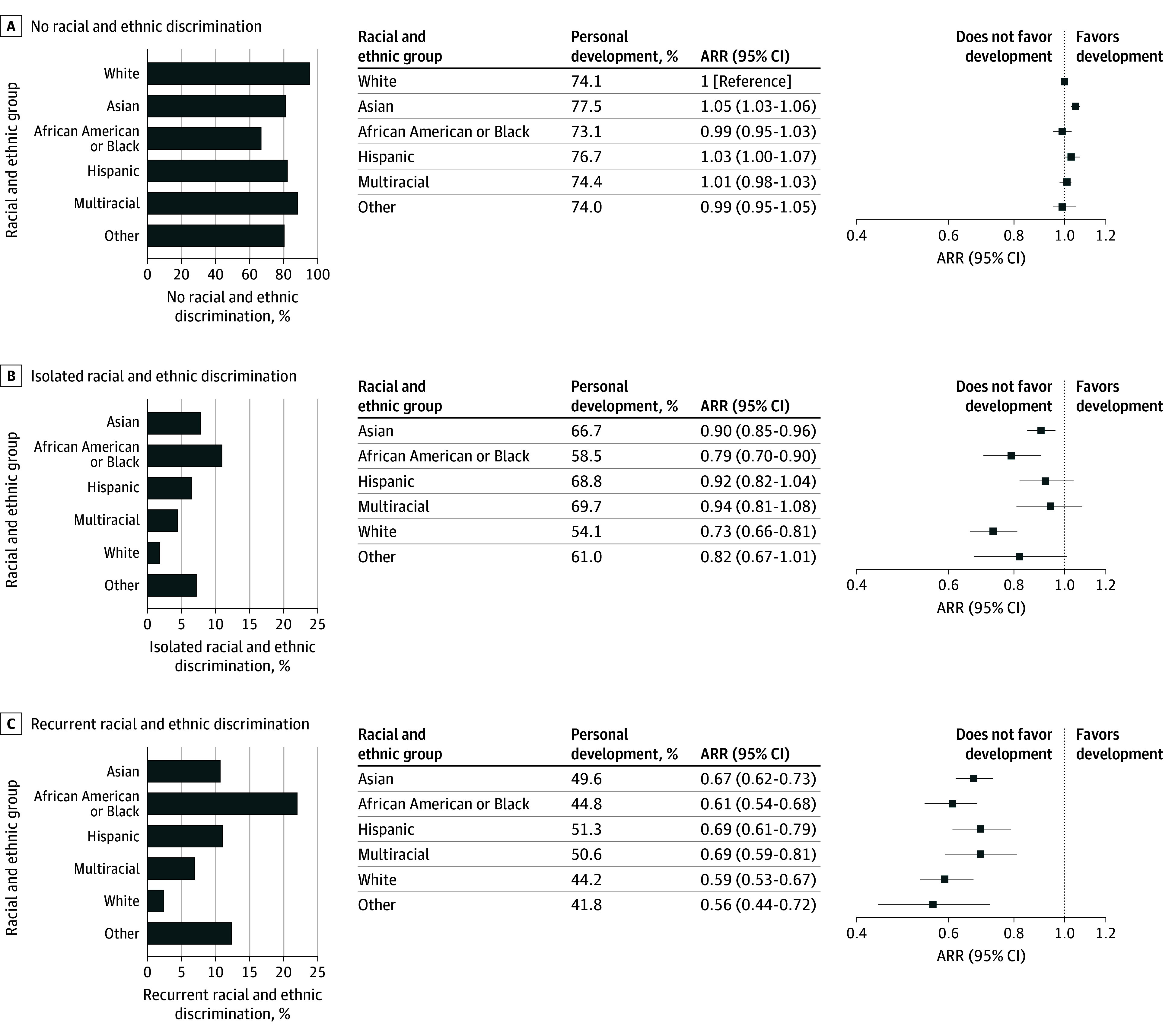
Prevalence of Racial and Ethnic Discrimination Among Medical School Students by Racial and Ethnic Group and the Association of Discrimination With Personal Development The bar graphs display the percentage of students from each racial and ethnic group who reported no racial and ethnic discrimination (A), isolated incidents of racial and ethnic discrimination (B), and recurrent racial and ethnic discrimination (C). The associated forest plots detail the sex-adjusted risk ratios (ARRs) for personal development, comparing each racial and ethnic group’s experience of isolated and recurrent racial and ethnic discrimination with White students with no such experiences.

**Figure 4.**
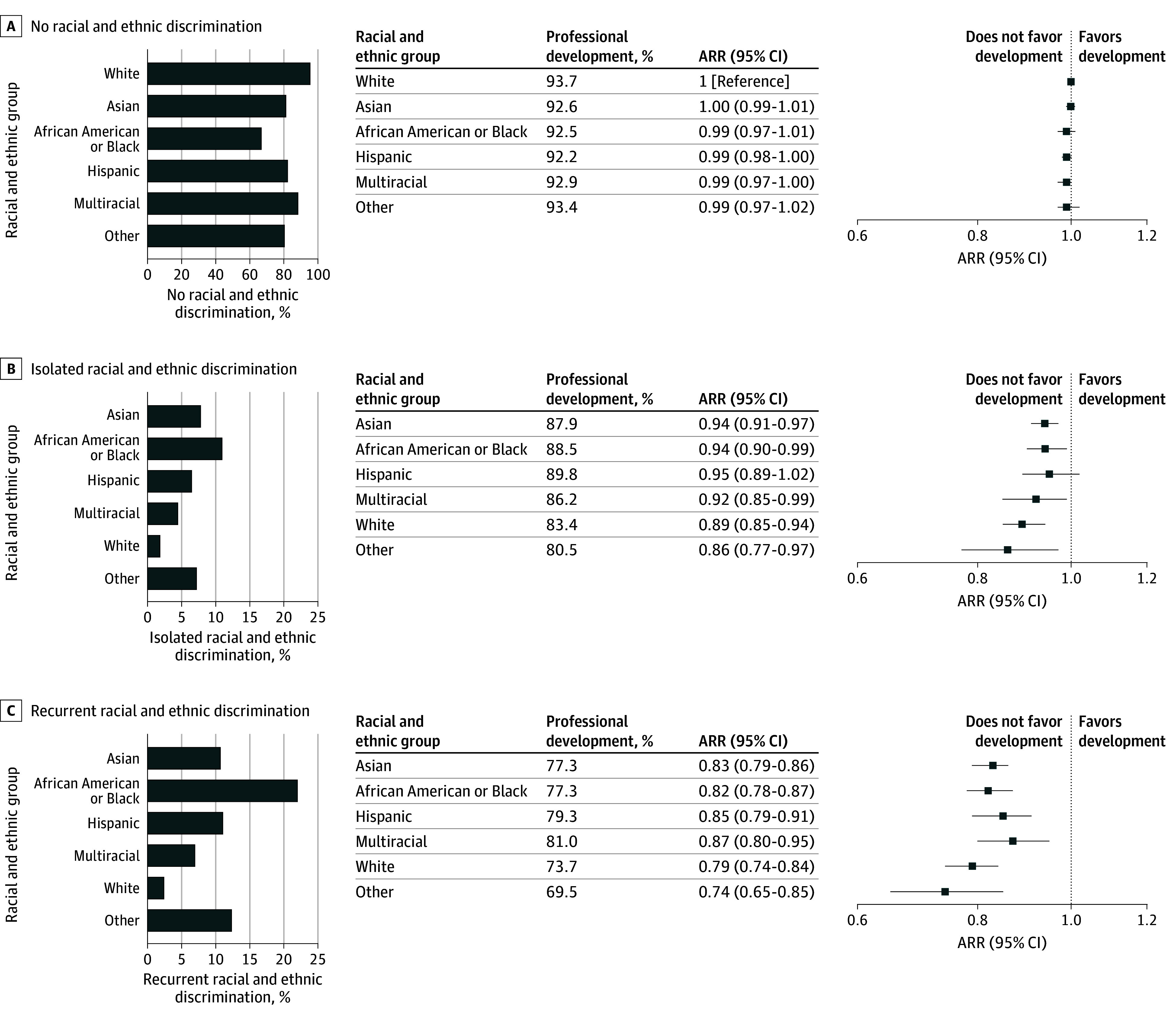
Prevalence of Racial and Ethnic Discrimination Among Medical School Students by Racial and Ethnic Group and the Association of Discrimination With Professional Development The bar graphs display the percentage of students from each racial and ethnic group who reported no discrimination (A), isolated incidents of racial and ethnic discrimination (B), and recurrent racial and ethnic discrimination (C). The associated forest plots detail the sex-adjusted risk ratios (ARRs) for professional development, comparing each racial and ethnic group’s experience of isolated and recurrent racial and ethnic discrimination with White students with no such experiences.

African American or Black students reported the highest percentage of isolated racial and ethnic discrimination at 11.0% (270 of 2458), while White participants reported the lowest at 1.9% (403 of 21 380) (χ^2^_5_ = 854.2; *P* < .001) (Figure 3 and Figure 4; eTable 1 in Supplement 1). The highest percentage of recurrent racial and ethnic discrimination was also reported by African American or Black students at 22.1% (543 of 2458), followed by Hispanic students (11.1% [271 of 2430]), Asian students (10.8% [841 of 7801]), multiracial students (6.9% [167 of 2404]), and White students (2.4% [505 of 21 380]) (χ^2^_5_ = 1955.6; *P* < .001). Conversely, White participants had the highest proportion of having never experienced racial and ethnic discrimination at 95.7% (20 472 of 21 380) (χ^2^_5_ = 2902.4; *P* < .001).

Among students who had never experienced racial and ethnic discrimination, there were no significant differences in the sex-adjusted likelihood of medical school fostering personal development by race and ethnicity (Figure 3). African American or Black students (ARR, 0.79 [95% CI, 0.70-0.90]), Asian students (ARR, 0.90 [95% CI, 0.85-0.96]), and White students (ARR, 0.73 [95% CI, 0.66-0.81]) experiencing isolated incidents of racial and ethnic discrimination were less likely to report that their medical school fostered their personal development compared with White students who had never experienced racial and ethnic discrimination. All ethnoracial groups experiencing recurrent discrimination reported significantly lower likelihood of their medical school fostering their personal development compared with White students who had never faced racial and ethnic discrimination. The ARRs for these groups were 0.61 (95% CI, 0.54-0.68) for African American or Black students, 0.67 (95% CI, 0.62-0.73) for Asian students, 0.69 (95% CI, 0.61-0.79) for Hispanic students, 0.69 (95% CI, 0.59-0.81) for multiracial students, and 0.59 (95% CI, 0.53-0.67) for White students.

Similar trends were observed for professional development. Among students who had never experienced racial and ethnic discrimination, there were no significant differences in the sex-adjusted likelihood of medical school fostering professional development by race and ethnicity (Figure 4). African American or Black students (ARR, 0.94 [95% CI, 0.90-0.99]), Asian students (ARR, 0.94 [95% CI, 0.91-0.97]), multiracial students (ARR, 0.92 [95% CI, 0.85-0.99]), and White students (ARR, 0.89 [95% CI, 0.85-0.94]) with isolated experiences of racial discrimination were less likely to report that their medical school fostered their professional development compared with White students who had never experienced discrimination. All ethnoracial groups experiencing recurrent racial and ethnic discrimination reported a significantly lower likelihood of their medical school fostering their professional development compared with White students who had never faced racial and ethnic discrimination.

## Discussion

This study reveals strong, graded, and consistent associations between experiences of racial and ethnic discrimination and lack of personal and professional development fostered by medical schools among their students, particularly highlighting the disproportionate influence on African American or Black students. Although existing research has outlined the presence of racial and ethnic discrimination in health professional education environments,^14^ to our knowledge, this is the first study to explore its role in shaping medical student PPIF.

### Racial and Ethnic Disparities in PPIF

The AAMC identifies personal and professional development as key competencies in medical education.^1^ Our study indicates that African American or Black students were less likely to report that their medical school fostered and nurtured their development as a person, and all other racial and ethnic minority groups underrepresented in medicine were less likely to report that their medical school fostered and nurtured their development as a professional compared with their White peers. Our finding that the discrimination faced by African American or Black and other students of racial and ethnic minority groups was associated with this discrepancy in PPIF^15,16^ provides insight regarding potential mechanisms underlying this disparity.

### Association of Discrimination With PPIF Across Racial and Ethnic Groups

Although prior research has focused primarily on the effect of discrimination on racial and ethnic minority groups, a major finding of this study is that experiences of racial and ethnic discrimination in medical education may be universally harmful to learners from all racial and ethnic backgrounds.^17,18,19^ As the frequency of the racial and ethnic discrimination experienced increased, students belonging to all racial and ethnic groups reported lower rates of personal and professional development. Experience of racial and ethnic discrimination may be associated with higher rates of burnout among medical students, a condition characterized by emotional exhaustion, depersonalization, and a diminished sense of personal accomplishment,^20^ which can contribute to depression,^6^ increased attrition from medical school,^8^ and, plausibly, an erosion of PPIF.

### Disproportionate Experience of Racial and Ethnic Discrimination Among African American or Black Students

This study revealed that both African American or Black and students of other racial and ethnic minority groups underrepresented in medicine disproportionately experience racial and ethnic discrimination compared with their White counterparts, who are overrepresented in the US physician workforce.^21,22,23,24,25^ Approximately one-third of African American or Black students experience racial and ethnic discrimination, and more than one-fifth face it recurrently.^26^ These experiences of discrimination may create a deleterious learning environment and have implications for the diversity of the physician workforce. A recent national study reported that students underrepresented in medicine are more likely to experience race and ethnicity–related microaggressions, leading to burnout and compromised learning.^27^ Not only African American or Black students^15^ but also African American or Black faculty members face more discrimination than their White counterparts.^28^ Among physicians, the experience of workplace discrimination is associated with diminished opportunities for career advancement, increased job turnover, career dissatisfaction, and contemplation of career change.^19,29^ Among medical students underrepresented in medicine, racial and ethnic discrimination may impede PPIF via its significant negative association with learning, academic performance, and well-being.^30^

### Association of Racial and Ethnic Discrimination With Personal vs Professional Development

Across all racial and ethnic groups, personal development may be more strongly associated with racial and ethnic discrimination than professional development. This discrepancy could be associated with the explicit focus of medical education on developing clinical competence and professional behaviors, as delineated by the Liaison Committee on Medical Education (LCME). The LCME accreditation guidelines do not explicitly address personal development; doing so could serve as another point of intervention to improve holistic PPIF among African American or Black students and students of other racial and ethnic minority groups underrepresented in medicine who experience the most racial and ethnic discrimination. Given the association of racial and ethnic discrimination with factors influencing personal development, including diminished well-being and mental health^31,32^ as well as decreased sense of agency and belonging,^32,33,34^ medical schools should consider efforts to foster personal development among African American or Black students and students of other racial and ethnic minority groups underrepresented in medicine. Students facing racial and ethnic discrimination may adapt by compartmentalizing these distressing experiences to uphold professionalism and emotional stability,^35,36^ potentially exacerbating the effect on their overall development throughout their careers.

### Implications

Our findings emphasize the imperative for systemic reforms in medical educational frameworks, to mitigate educational disparities and foster holistic development among students underrepresented in medicine. Specifically, we propose considering the integration of PPIF as a critical equity metric and the formal inclusion of PPIF parity across students of all ethnoracial backgrounds in the LCME accreditation standards. In addition, educators, institutions, and entities such as the LCME should consider antiracism initiatives as not only benefitting minoritized groups but as enhancing the development of all students. Promoting a sense of belonging in medicine for all students by addressing unconscious biases and transforming academic medicine environments to recognize, hear, and value diverse contributions may positively influence PPIF in medical school.^37,38^ Medical schools could also explore tools to achieve a climate of equity and inclusion within the learning environment.

### Limitations

This study has some limitations. The complexities of self-reporting race and ethnicity, influenced by subjective and historical experiences and further compounded by limitations in questionnaires, such as the lack of a Middle Eastern and North African category in AAMC surveys for our data years, must be considered when interpreting our results. Some multiracial students may identify more with one race or ethnicity over another, leading to varied self-identifications and experiences of discrimination. Furthermore, our study likely underestimates the true scale of racism and discrimination encountered by African American or Black students and other students underrepresented in medicine, due to higher attrition rates among students underrepresented in medicine, indicating a potential survival bias in analyses conducted at certain time points, such as graduation from medical school.^39^ In addition, experience of racial and ethnic discrimination may potentially be underreported due to response bias.^40^ Finally, we acknowledge that while our data suggest strong associations between the experience of racial and ethnic discrimination and diminished PPIF, causality must not be presumed.

## Conclusions

This cross-sectional study of US allopathic medical students found an association between racial and ethnic discrimination and the effectiveness of medical schools in nurturing students’ PPIF. African American or Black students were less likely than their White counterparts to feel that medical school contributed to their development as a person and physician. In addition, an increase in the frequency of racial and ethnic discrimination was associated with a decreased likelihood that their medical school supported their PPIF, reported by students of all racial and ethnic backgrounds. Significantly, African American or Black students and students of other racial and ethnic minority groups reported experiencing racial and ethnic discrimination more frequently than White students. More work is needed to elucidate the mechanisms by which racial and ethnic discrimination may be associated with PPIF in medical school and to design multilevel interventions to eradicate racial and ethnic discrimination and support the holistic development of all students, with a particular focus on those students underrepresented in medicine.

## Notes

Supplement 1. **eTable 1.** Frequency of Racial Discrimination Experienced by Race/Ethnic Subgroups **eTable 2.** The Association of Sex With Personal and Professional Development After Adjusting for Race/Ethnicity

Supplement 2.
Data Sharing Statement
