# Supplementary material for: Racial and Ethnic Discrimination and Medical Students’ Identity Formation
Source: JAMA Netw Open. 2024 Oct 16;7(10):e2439727. doi: 10.1001/jamanetworkopen.2024.39727 (PMC11581615; doi:10.1001/jamanetworkopen.2024.39727)
Supplement: Supplement 1. — eTable 1. Frequency of Racial Discrimination Experienced by Race/Ethnic Subgroups eTable 2. The Association of Sex With Personal and Professional Development After Adjusting for Race/Ethnicity [file jamanetwopen-e2439727-s001.pdf]

Supplementary Online Content

Venkataraman S, Nguyen M, Chaudhry S, et al. Racial and ethnic discrimination and medical students' identity formation. *JAMA Netw Open.* 2024;7(10):e2439727. doi:10.1001/jamanetworkopen.2024.39727

eTable 1. Frequency of Racial Discrimination Experienced by Race/Ethnic Subgroups
eTable 2. The Association of Sex With Personal and Professional Development After Adjusting for Race/Ethnicity

This supplementary material has been provided by the authors to give readers additional information about their work.

© 2024 Venkataraman S et al. *JAMA Network Open.*

eTable 1. Frequency of Racial Discrimination Experienced by Race/Ethnic Subgroups

Racial/Ethnic Subgroup	No experience of racial discrimination Participants, N (%)	Isolated experience of racial discrimination Participants, N (%)	Recurrent experience of racial discrimination Participants, N (%)	Total N (%)
African American/Black	1 645 (66.9)	270 (11.0)	543 (22.1)	2 458
Asian	6 346 (81.3)	614 (7.9)	841 (10.8)	7 801
Hispanic	2 002 (82.4)	157 (6.5)	271 (11.1)	2 430
Multiracial	2 129 (88.6)	108 (4.5)	167 (6.9)	2 404
white	20 472 (95.7)	403 (1.9)	505 (2.4)	21 380
Other	914 (80.4)	82 (7.2)	141 (12.4)	1 137
Total, N	33 508	1634	2468	37610

© 2024 Venkataraman S et al. *JAMA Network Open.*

eTable 2. The Association of Sex With Personal and Professional Development After Adjusting for Race/Ethnicity

	aRR of Personal Development (95% CI)	P-value	aRR of Professional Development (95% CI)	P-value
Males	Reference	-	Reference	-
Females	0.97 (0.96-0.98)	<0.001	1.01 (1.01-1.02)	<0.001

© 2024 Venkataraman S et al. *JAMA Network Open.*
