# Supplementary material for: Racial and Ethnic Discrimination and Medical Students’ Identity Formation
Source: JAMA Netw Open. 2024 Oct 16;7(10):e2439727. doi: 10.1001/jamanetworkopen.2024.39727 (PMC11581615; doi:10.1001/jamanetworkopen.2024.39727)
Supplement: Supplement 2. — Data Sharing Statement [file jamanetwopen-e2439727-s002.pdf]

Data Sharing Statement

Venkataraman. Racial and Ethnic Discrimination and Medical Students' Identity Formation. *JAMA Netw Open*. Published October 16, 2024. doi:10.1001/jamanetworkopen.2024.39727

Data
Data available: No
